# MiR-27a-3p suppresses cerebral ischemia-reperfusion injury by targeting FOXO1

**DOI:** 10.18632/aging.202866

**Published:** 2021-04-19

**Authors:** Wenyu Li, Qiongbin Zhu, Xiaoyan Xu, Xingyue Hu

**Affiliations:** 1 Department of Neurology, Sir Run Run Shaw Hospital, Zhejiang University School of Medicine, Hangzhou 133000, Zhejiang, China

**Keywords:** CI/R, miR-27a-3p, FOXO1, cell injury

## Abstract

Cerebral ischemia-reperfusion (CI/R) injury is a serious complication when treating patients experiencing ischemic stroke. Although the microRNA miR-27a-3p reportedly participates in ischemia/reperfusion (I/R) injury, its actions in CI/R remain unclear. To mimic CI/R *in vitro*, HT22 cells were subjected to oxygen glucose deprivation/reoxygenation (OGD/R). The results indicate that OGD inhibited growth and induced apoptosis among HT22 cells. The apoptosis was accompanied by increases in activated caspases 3 and 9 and decreases in Bcl-2. Oxidative stress was also increased, as indicated by increases in ROS and malondialdehyde and decreases in glutathione and superoxide dismutase. In addition, OGD induced G1 arrest in HT22 cells with corresponding upregulation of FOXO1 and p27 Kip1, suggesting the cell cycle arrest was mediated by FOXO1/p27 Kip1 signaling. Notably, FOXO1 was found to be the direct target of miR-27a-3p in HT22 cells. MiR-27a-3p was downregulated in OGD/R-treated HT22 cells, and miR-27a-3p mimics partially or entirely reversed all of the *in vitro* effects of OGD. Moreover, miR-27a-3p agomir significantly alleviated the symptoms of CI/R *in vivo* in a rat model of CI/R. Thus, MiR-27a-3p appears to suppress CI/R injury by targeting FOXO1.

## INTRODUCTION

Each year, cerebral vascular rupture or ischemia, or stroke, results in over 40 million disabilities worldwide [1]. Ischemic stroke is currently a leading cause of disability in adults [2]. The main treatment for ischemic stroke is immediate restoration of the blood supply, though this may exacerbate the brain damage due to cerebral ischemia-reperfusion injury (CI/R) [3]. At present, the beneficial effects of drug and surgical treatments for CI/R are limited, and there is an urgent need to find new strategies for the treatment of CI/R injury.

MicroRNAs (miRNAs) are non-coding RNAs involved in the development of multiple diseases, including CI/R [4–6]. For instance, miR-532-3p downregulation is known to aggravate CI/R injury by targeting NOX2 [7], and Zuo et al. showed that miR-652 could protect against CI/R injury through suppression of NOX2 [8]. In addition, MiR-27a-3p is reportedly involved in the progression of ischemia/reperfusion (I/R) injury [9], though the biological function of miR-27a-3p in CI/R remains unclear.

Forkhead box class O1 (FOXO1) is a transcription factor which is involved in cellular process [10]. In addition, some reports have indicated that FOXO1 can regulate the progression of I/R injury. For instance, Chen YQ et al found that FOXO1 could alleviate the progression of myocardial ischemia-reperfusion injury [11]; Wang D et al indicated that FOXO1 downregulation could inhibit the progression of renal ischemia-reperfusion injury [12]. Meanwhile, FOXO1 is known to promote the development of CI/R [13]. However, the correlation between miR-27a-3p and FOXO1 in CI/R remains unclear.

In the present study, we investigated the role of miR-27a-3p during the CI/R process with the aim of providing new perspectives that would contribute to the development of improved treatment strategies for patients with CI/R. We hope this research would shed new lights on exploring the new strategies for the treatment of CI/R.

## RESULTS

### OGD-induced inhibition of HT22 cell growth is significantly reversed by miR-27a-3p mimics

**Figure 1:**
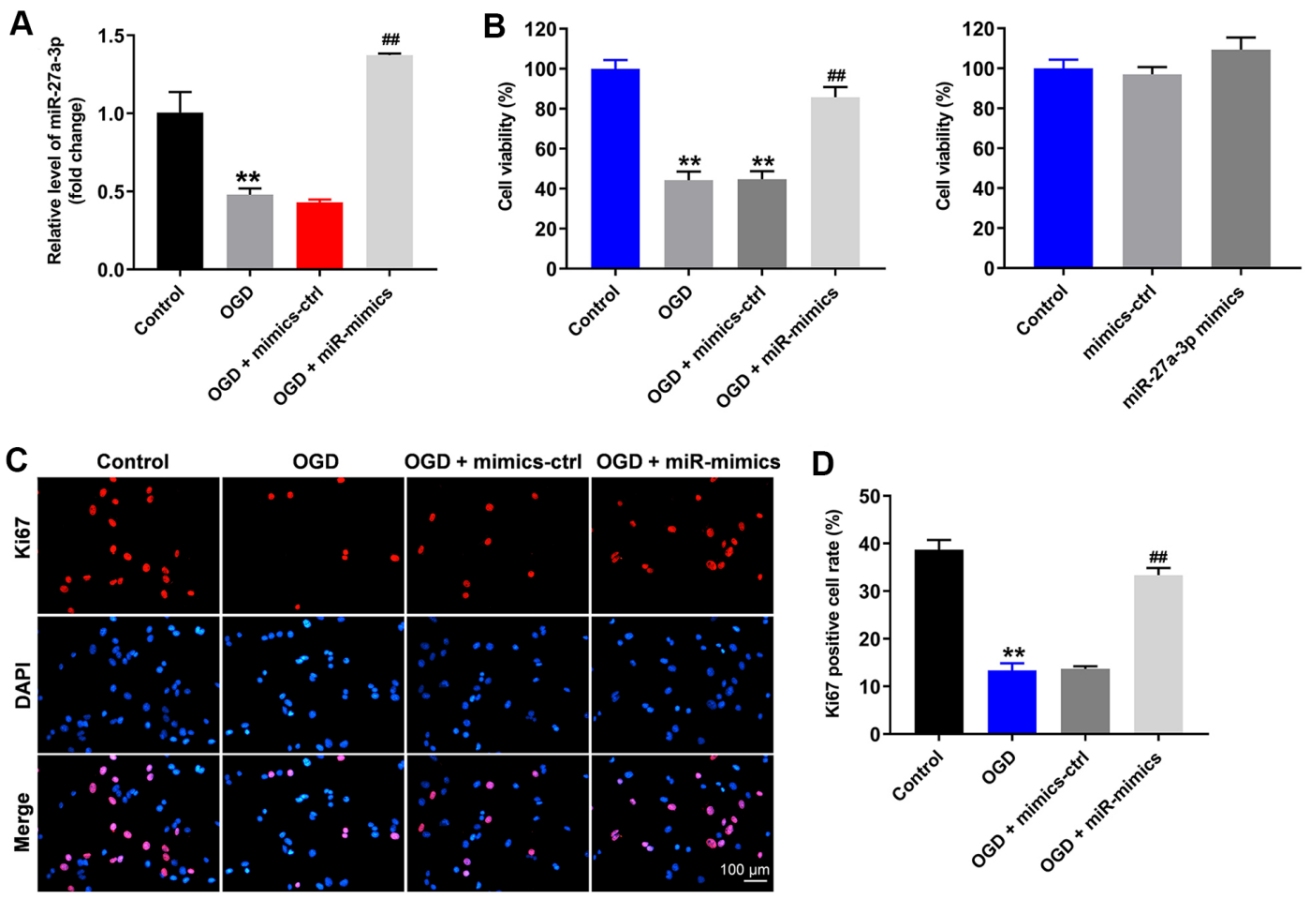
**OGD-induced HT22 cell growth inhibition is significantly reversed by miR-27a-3p mimics.** HT22 cells were transfected with mimics-control or miR-27a-3p mimics and then treated with OGD. (**A**) Expression of miR-27a-3p in HT22 cells detected with q-PCR. (**B**) Viability of HT22 cells assessed with CCK-8 assays. (**C**) Ki67 staining (red) showing proliferation of HT22 cells. Nuclei were counterstained with DAPI (blue). (**D**) Rate of Ki67 positivity. ^**^P<0.01 vs. control. ^##^P<0.01 vs. OGD.

To mimic CI/R injury *in vitro*, the oxygen-glucose deprivation (OGD) cell model was established. As indicated in Figure 1A, OGD significantly inhibited miR-27a-3p expression in HT22 cells, and this inhibitory effect was reversed by miR-27a-3p mimics. In addition, the viability of HT22 cells was significantly reduced by OGD, while this effect, too, was significantly reversed by miR-27a-3p mimics (Figure 1B). Meanwhile, miR-27a-3p mimics alone limitedly affected the viability of HT22 cells (Figure 1B). Consistent, with those findings, the antiproliferative effect of OGD on HT22 cells was significantly reversed by miR-27a-3p mimics (Figure 1C, 1D). All of these data suggested that OGD-induced HT22 cell growth inhibition is reversed by miR-27a-3p mimics.

### OGD-induced cell apoptosis is reversed by miR-27a-3p mimics

**Figure 2:**
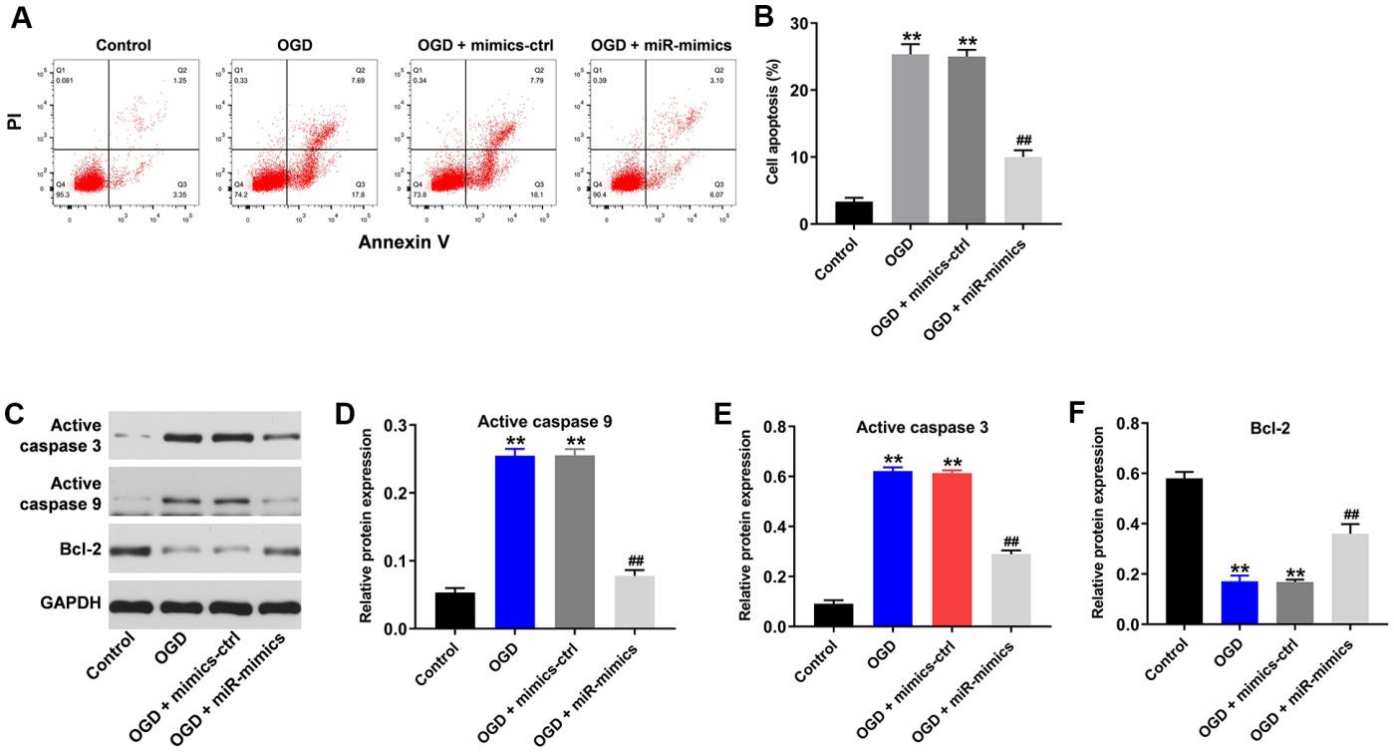
**OGD-induced cell growth inhibition is reversed by miR-27a-3p mimics.** (**A**) FACS analysis of cell apoptosis. (**B**) The rate of apoptosis among HT22 cells. (**C**) Western blotting showing levels of active caspase 3, active caspase 9 and Bcl-2 in HT22 cells. (**D**–**F**) Relative protein expression levels of active caspase 3 (**D**), active caspase 9 (**E**) and Bcl-2 (**F**) normalizing to GAPDH. ^**^P<0.01 vs. control. ^##^P<0.01 vs. OGD.

Using flow cytometry to detect cell apoptosis, we observed that OGD clearly induced apoptosis in HT22 cells (Figure 2A, 2B). Consistent with the increased apoptosis, levels of active caspase 3 and active caspase 9, two pro-apoptosis enzymes, were significantly increased in HT22 cells in the presence of OGD (Figure 2C–2E). Conversely, levels of the anti-apoptotic protein (Bcl-2) was significantly decreased in HT22 cells in the presence of OGD (Figure 2C, 2F). All of these effects were reversed in the presence of miR-27a-3p mimics (Figure 2A–2F).

### MiR-27a-3p reverses OGD-induced injury in HT22 cells

**Figure 3:**
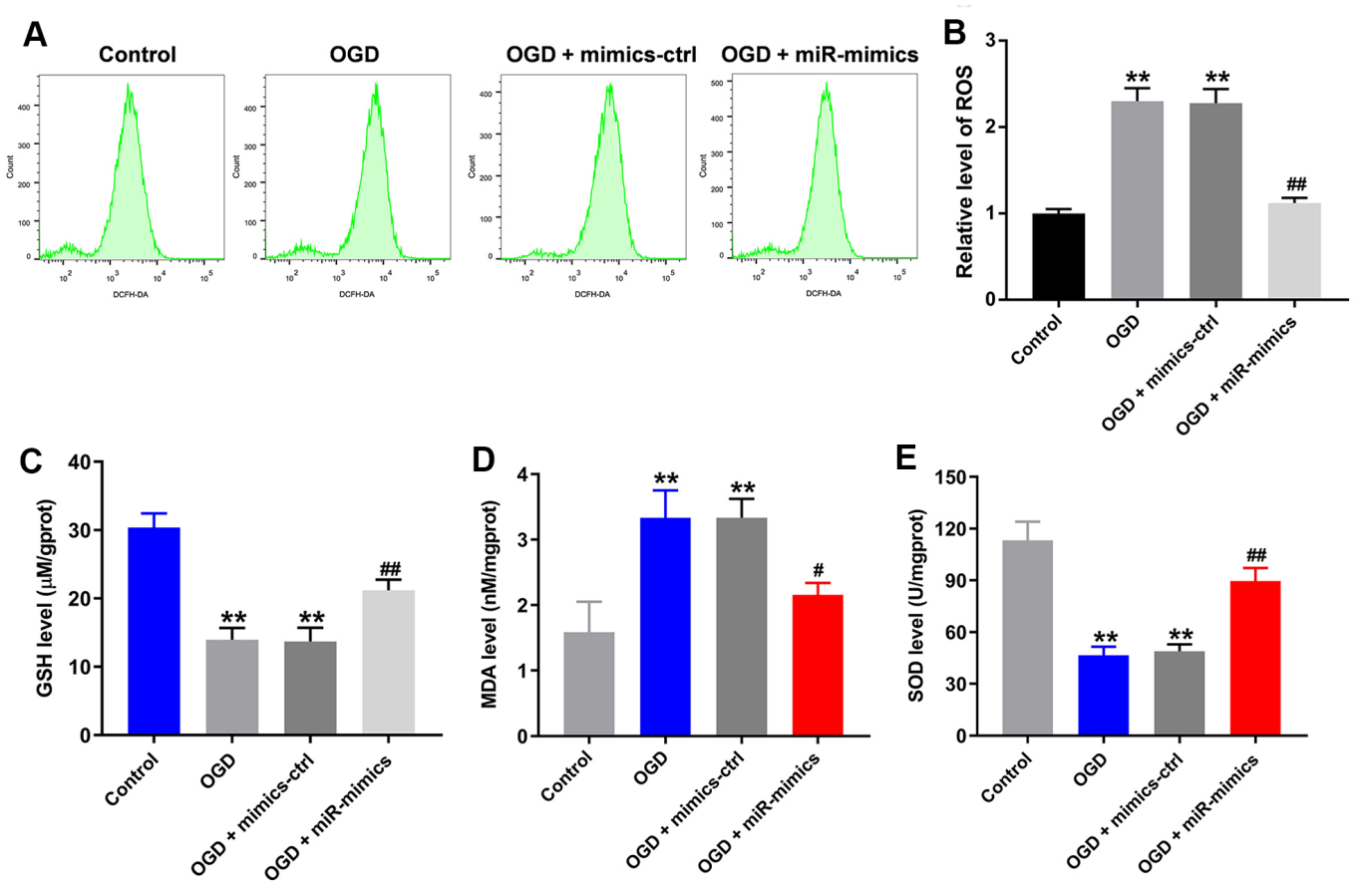
**MiR-27a-3p protects against OGD-induced injury in HT22 cells.** (**A**) FACS analysis of ROS level in HT22 cells. (**B**) Relative levels of ROS. (**C**–**E**) Glutathione (GSH) (**C**), malondialdehyde (MDA) (**D**) and superoxide dismutase (SOD) levels (**E**) in detected in supernatants of HT22 cells with ELISAs. ^**^P<0.01 vs. control. ^#^P<0.05, ^##^P<0.01 vs. OGD.

Using flow cytometry to detect levels of reactive oxygen species (ROS) in HT22 cells, we found that OGD upregulated ROS levels in HT22 cells (Figure 3A, 3B). OGD also significantly reduced the levels of the antioxidants glutathione and superoxide dismutase and increased the levels of malondialdehyde, a marker of oxidative stress, in the supernatants of HT22 cells (Figure 3C–3E). Again, all of these effects were at least partially reversed in the presence of miR-27a-3p mimics (Figure 3A–3E). Thus, miR-27a-3p mimics clearly suppressed OGD-mediated oxidative injury in HT22 cells.

### MiR-27a-3p directly targets FOXO1

**Figure 4:**
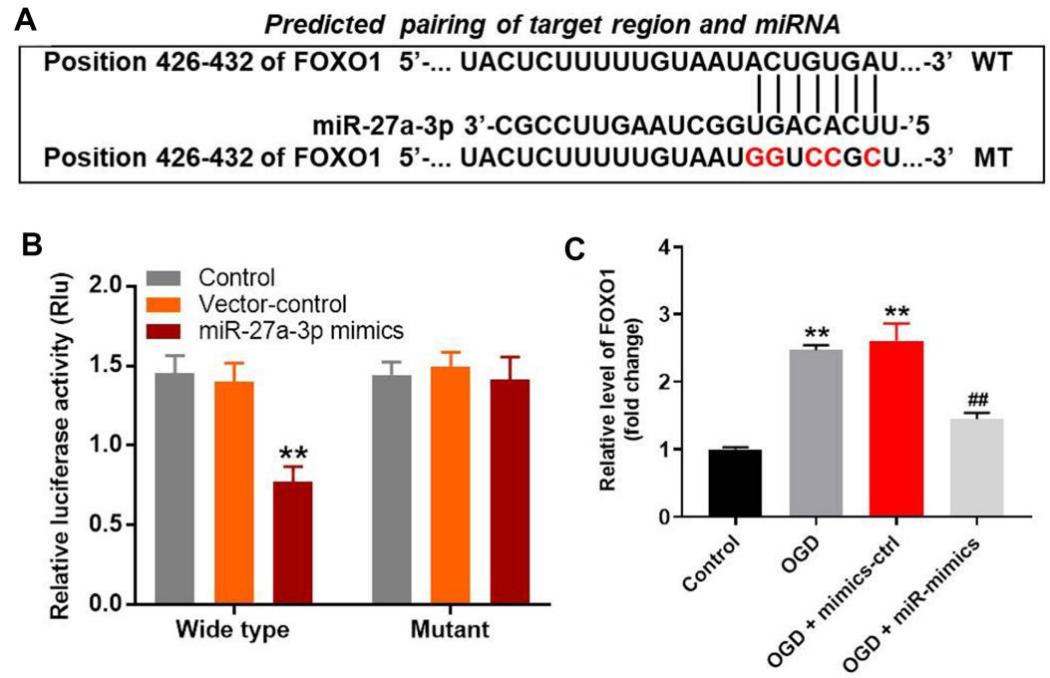
**MiR-27a-3p directly targets FOXO1 in HT22 cells.** (**A**) Gene structure of *FOXO1* at position 426-432 indicates the predicted target binding site of miR-27a-3p in its 3'UTR. (**B**) Luciferase activity measured after co-transfecting with wild-type or mutant FOXO1 3′-UTR plasmid and miR-27a-3p mimics into HT22 cells. The results were normalized to Renilla luciferase activity. (**C**) RT-qPCR analysis of FOXO1 expression in HT22 cells. ^**^P<0.01 vs. control. ^##^P<0.01 vs. OGD.

The targetscan (http://www.targetscan.org/vert_71/), miRDB (http://www.mirdb.org/) and miRWalK (http://zmf.umm.uni-heidelberg.de/apps/zmf/mirwalk/micrornapredictedtarget.html) on-line tools were used to explore the gene targeted by miR-27a-3p. As shown in Figure 4A *FOXO1* was the likely target of miR-27a-3p. This was confirmed by dual luciferase assays, which showed the suppression of *FOXO1* transcription by miR-27a-3p (Figure 4B). Additionally, RT-qPCR demonstrated that miR-27a-3p mimics significantly reversed OGD-induced increases in *FOXO1* gene expression in HT22 cells (Figure 4C).

### MiR-27a-3p reverses OGD-induced G1 arrest in HT22 cells via mediation of FOXO1/p27 Kip1 axis

**Figure 5:**
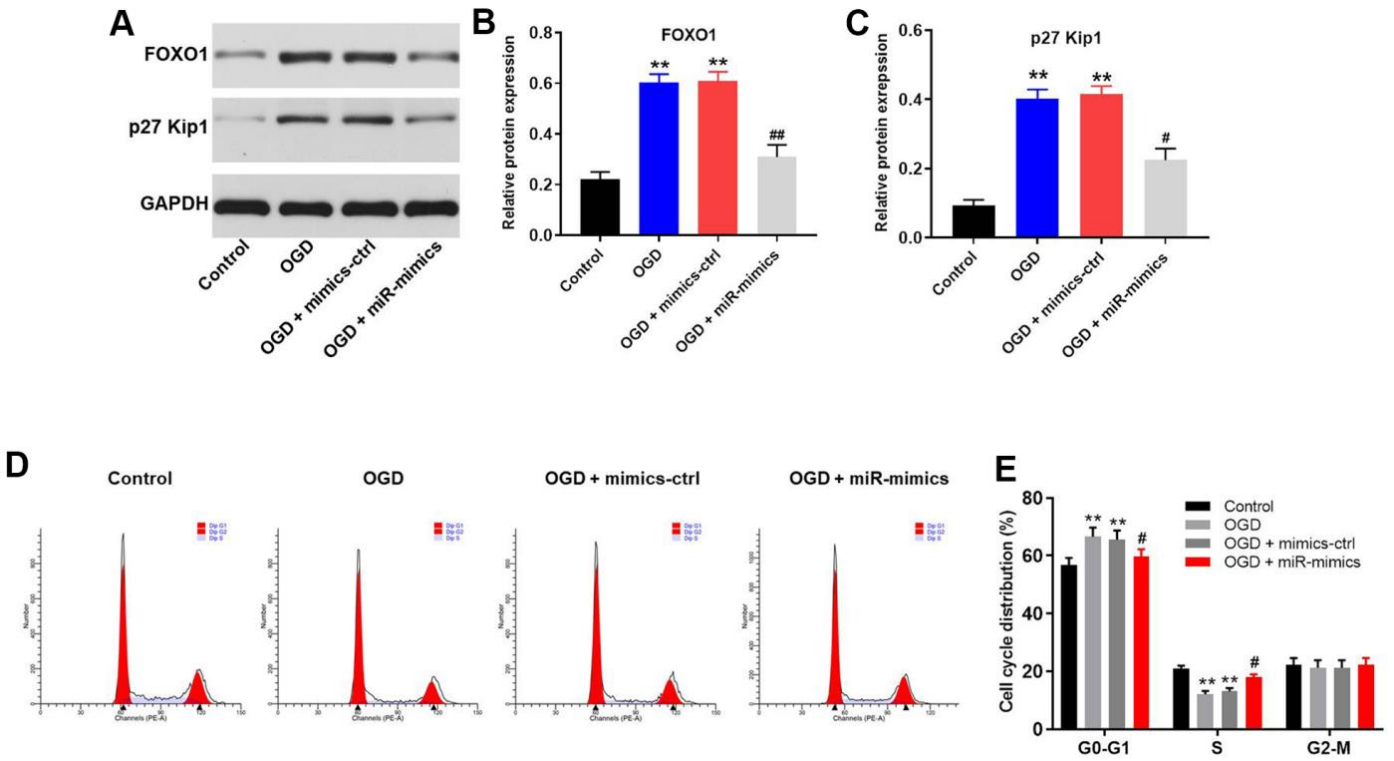
**MiR-27a-3p reverses OGD-induced G1 arrest in HT22 cells via mediation of FOXO1/p27 Kip1 axis.** (**A**) Western blotting showing the protein expression of FOXO1 and p27 Kip1 in HT22 cells. (**B**, **C**) Relative expression levels of FOXO1 (**B**) and p27 Kip1 (**C**) normalized to GAPDH. (**D**) FACS analysis of HT22 cell cycling after PI staining. (**E**) Cell cycle profile showing distribution of cells in G0/G1, S or G2 phase. ^**^P<0.01 vs. ^#^P<0.05, ^##^P<0.01 vs. OGD.

We used western blotting to explore the mechanism by which OGD mediates the progression of CI/R *in vitro*. As shown in Figure 5A–5C, OGD upregulated protein expression of FOXO1 and p27 Kip1, the cell cycle mediator [14], in HT22 cells, while this phenomenon was suppressed in the presence of miR-27a-3p mimics. Moreover, flow cytometric analysis confirmed that OGD induced G1 arrest in HT22 cells, while overexpression of miR-27a-3p partially reversed the effect of OGD on cell cycle distribution (Figure 5D, 5E). These results suggest that OGD induces G1 arrest in HT22 cells via FOXO1/p27 Kip1 signaling, and that effect is reversed by miR-27a-3p.

### MiR-27a-3p agomir significantly attenuates the symptom of CI/R *in vivo*

**Figure 6:**
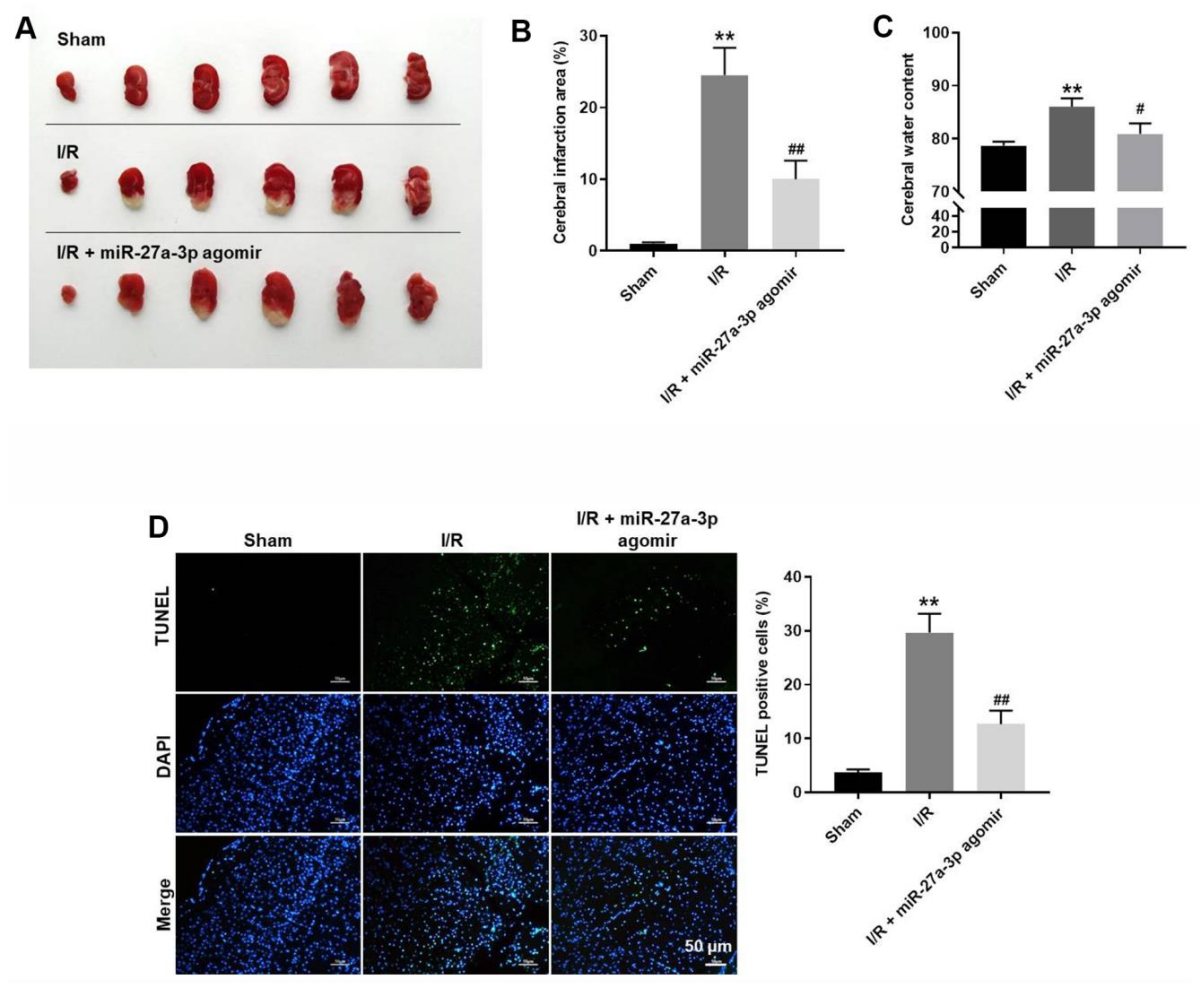
**MiR-27a-3p agomir significantly alleviates the symptoms of CI/R *in vivo*.** (**A**) Images of brain tissues from rats. (**B**) Calculated cerebral infraction area. (**C**) Cerebral water content. (**D**) TUNEL showing apoptosis within brain tissues. ^**^P< 0.01 vs. sham. ^#^P<0.05, ^##^P<0.01 vs. I/R.

**Figure 7:**
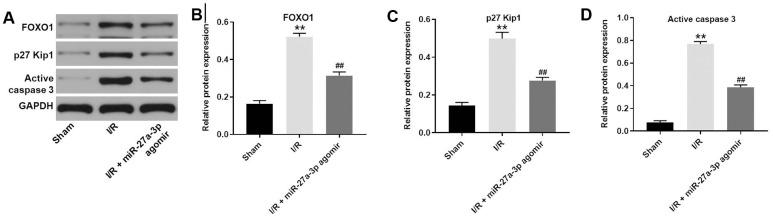
**MiR-27a-3p agomir inhibits the expression of FOXO1, p27 Kip1 and active caspase 3 in brain tissues of rats.** (**A**) Protein levels of FOXO1, p27 Kip1 and active caspase 3 in brain tissue from rats. (**B**–**D**) Relative expression levels of FOXO1 (**B**), p27 Kip1 (**C**) and active caspase 3 (**D**) normalized to GAPDH levels. ^**^P<0.01 vs. sham. ^##^P<0.01 vs. I/R.

Finally, a rat model of CI/R was established to explore the effect of miR-27a-3p on CI/R progression *in vivo*. As indicated in Figure 6A, 6B, miR-27a-3p agomir significantly reduced cerebral infraction area in CI/R rats. In addition, miR-27a-3p agomir reversed CI/R-induced increases of cerebral water content and the TUNEL positivity rate (Figure 6C, 6D). Consistent with our *in vitro* findings, CI/R induced increases in FOXO1, p27 Kip1 and active caspase 3 in brain tissues of the model rats, but those effects were significantly reversed by miR-27a-3p agomir (Figure 7A–7D). It thus appears that miR-27a-3p agomir significantly attenuates CI/R responses *in vivo*.

## DISCUSSION

It was recently reported that the miRNAs were involved in progression of CI/R [7, 15, 16]. In the present study, we found that the progression of CI/R is significantly suppressed by miR-27a-3p mimics. This is the first study to identify potential actions of miR-27a-3p in CI/R and to show that miR-27a-3p could act as a suppressor in CI/R. An earlier study found that downregulation of miR-27a-3p attenuated high glucose-triggered podocyte apoptosis, fibrosis and inflammation by targeting tissue inhibitor of metalloproteinase 3 (TIMP3) [17]. TIMP3 overexpression reportedly inhibits the cell apoptosis [18]. This suggests the net effect of miR-27a-3p may be the product of its diverse functions.

MiRNAs exert their effects by suppressing expression of their target genes [19, 20]. In the present study, luciferase reporter assays revealed *FOXO1* to be a direct target gene of miR-27a-3p in CI/R. In addition, miR-27a-3p apparently suppressed FOXO1/p27 Kip1 signaling. FOXO1 is a member of the FOX protein family, which is thought play role in cell apoptosis [21]. On the other hand, p27 Kip1 is a cell cycle regulator first identified as a cyclin-dependent kinase antagonist [22]. It has been reported that E2F1-Ror2 signaling promotes G1/S phase transition in bFGF-stimulated NIH/3T3 fibroblasts via p27 Kip1 [23]. Additionally, Yang et al. reported that pentoxifylline inhibits the progression of fibrosis by modulating FOXO1/p27 Kip1 signaling [24]. We found that miR-27a-3p agomir suppressed levels of p27 Kip1, suggesting that miR-27a-3p suppresses CI/R progression via its inhibitory effects on FOXO1/p27 Kip1 signaling. It was also previously reported that miR-27a-3p mediates the development of retinoblastoma via targeting PEG10 [25]. However, this effect does not appear to be involved in its protective effects again CI/R.

Our *in vitro* experiments indicated that upregulation of miR-27a-3p significantly inhibited the apoptotic effects of OGD on HT22 cells by reducing levels of activated caspases 3 and 9, which are known to promote apoptosis [26], and increasing levels of Bcl-2, a known anti-apoptotic protein [27]. Zhang et al. reported that fibrauretine in combination with ginsenosides significantly reduced the progression of Alzheimer's disease through upregulation of Bcl-2 and downregulation of activated caspases 3 and 9 [28]. Our findings are consistent to these earlier ones, in that miR-27a-3p mimics suppressed apoptosis among OGD-treated HT22 cells through its effects on caspases 3 and 9.

According to Zhao XR et al [9], miR-27a-3p could aggravate renal ischemia/reperfusion injury through targeting Grb2. In contrast, our data revealed that miR-27a-3p could suppress cerebral ischemia-reperfusion injury by targeting FOXO1. In our study, FOXO1 was found to be targeted by miR-27a-3p. FOXO1 upregulation could inhibit the cell growth [11], while Grb2 can promote the cell proliferation [29]. Thus, the different function between FOXO1 and Grb2 might result in this discrepancy.

Two important limitations to this study are as follows. (1) This study focused only on the effect of miR-27a-3p on cell growth-related proteins. (2) Only one mRNA was found to be targeted by miR-27a-3p. Clearly additional investigations are needed in the future.

In summary, upregulation of miR-27a-3p significantly reduced CI/R injury by targeting *FOXO1*. While there is much work yet to be done, we suggest our findings could serve as the basis for future investigations into new approaches to the treatment of CI/R.

## MATERIALS AND METHODS

### Cell culture and establishment of *in vitro* OGD model

HT22 cells were obtained from the American Type Culture Collection (Manassas, VA, USA) and cultured in an incubator with Dulbecco’s modified Eagle medium (DMEM) supplemented with 10% fetal bovine serum (FBS) and 100 U/mL penicillin and streptomycin at 37° C under 5% CO_2_/95% O_2_. For oxygen-glucose deprivation (OGD) treatment, HT22 cells were placed first in deoxygenated glucose-free medium and then incubated in a hypoxic vessel for 4 h at 37° C under 95% N_2_/5% CO_2_. The cells were then transferred to DMEM supplemented with high glucose and 10% FBS under normoxic conditions (5% CO_2_) at 37° C for 24 h, as previously described [30].

### Cell transfection

HT22 cells were cultured in DMEM supplemented with 10% FBS, streptomycin and penicillin (100 U/mL) at 37° C under 5% CO_2_. The cells were transfected for 4 h with mimics-control or miR-27a-3p mimics using Lipofectamine 2000. The cells were then washed in warmed medium and incubated for 24 h to establish an *in vitro* model. Mimics-control and miR-27a-3p mimics were provided with RiboBio (Guangzhou, China).

### Quantitative real time polymerase chain reaction (RT-qPCR)

Total RNA was extracted from HT22 cells using TRIzol reagent (Thermo Fisher Scientific) and then reverse transcribed into cDNA using the PrimeScript RT reagent kit (TaKaRa, Otsu, Shiga, Japan). RT-qPCR was performed using a SYBR® Premix Ex Taq™ II kit (TaKaRa Bio, Otsu, Shiga, Japan) on a 7900HT system (Applied Biosystems, CA, USA). The PCR protocol entailed incubation at 60° C for 1 min and 90° C for 15 min, followed by 40 cycles at 90° C for 15 s and 55° C for 60 s. The primers used were from GeneCreate Biological Engineering Co., Ltd (Wuhan, China) and were as follows: for MiR-27a-3p, 5’-TCACAGTGGCTAAGTTCCGC-3’ (forward) and 5’-CTCAACTGGTGTCGTGGAGTC-3’. (reverse); for FOXO1, 5’-CAAAATGATGAACCCCAGCTC-3’ (forward) and 5’-CATCCTACCATAGCCATTGCAG-3’ (reverse); for GAPDH: 5’-TGAAGGGTGGAGCCAAAAG-3’ (forward) and 5’-AGTCTTCTGGGTGGCAGTGAT-3’ (reverse) and for U6, 5’-CTCGCTTCGGCAGCACAT-3’ (forward) and 5’-AACGCTTCACGAATTTGCGT-3’ (reverse). The relative mRNA levels were quantified using the 2^−ΔΔCt^ method and normalized to GAPDH or U6.

### Immunofluorescence

HT22 cells were plated onto a 96-well plate at the density of 5.0×10^3^ cells/well. After incubation, the cells were transfected for 72 h with NC or miR-27a-3p mimics. The cells were then fixed in 4% paraformaldehyde and incubated first with anti-Ki67 antibody (1:100, Abcam Cambridge, MA, USA) overnight at 4° C and then with anti-rabbit IgG secondary antibody (1:1000, Abcam) for 1 h at room temperature. The nuclei was stained with DAPI (Beyotime, Shanghai, China). Finally, the labeled cells were observed under a fluorescence microscope (Olympus BX53 Tokyo, Japan).

### CCK-8 assay

HT22 cells (5.0×10^3^ cells/well) were treated for 48 h with OGD, OGD + mimics-control, or OGD + miR-27a-3p mimics, after which 10 μl of CCK-8 reagents (Beyotime, Shanghai, China) were added into each well, and the plate was incubated for an additional 2 h at 37° C. The absorbance at 450 nm was then measured using a microplate reader (Bio-Rad, Hercules, CA, USA).

### Cell apoptosis analysis

After trypsinizing HT22 cells and resuspending them in binding buffer, they were stained with 5 μl annexin V-FITC (BD Biosciences, Franklin Lake, NJ, USA) and propidium iodide (PI; BD Biosciences, Franklin Lake, NJ, USA) for 30 min in the dark at 37° C. Fluorescence-activated cell sorting (FACScan™; BD Biosciences, Franklin Lake, NJ, USA) was then applied to analyze the apoptosis rate using Image J software (BD Biosciences, Franklin Lake, NJ, USA).

### ROS detection

HT22 cell suspensions were collected and supplemented with the ROS probe DCFDA (Beyotime, Shanghai, China) as previously described [31]. After 20 min of incubation, cells were centrifuged at 300 *g*, washed with PBS and resuspended. Relative ROS levels were measured using FACS (FACScan™; BD Biosciences, Franklin Lake, NJ, USA).

### Enzyme-linked immunosorbent assay (ELISA)

Supernatants were collected from HT22 cells, and levels of GSH, MDA and SOD were measured using ELISA kits (ELK Biotech, Wuhan, China) according to the manufacturer’s protocols.

### Cell cycle detection

HT-22 cells were harvested and counted with a hemocytometer. They were then fixed, permeabilized, stained with 5 μl propidium iodide (PI) and analyzed using FACS with a FACSCalibur (BD Biosciences, Franklin Lake, NJ, USA). The data were quantified using ModFit and FlowJo software.

### Western blot analysis

HT22 cells or brain tissues were lysed in RIPA lysis buffer (KeyGEN, Nanjing, China), the protein concentrations in the lysates were determined using a BCA Assay kit (Solar Life Science, Beijing, China). Equal amounts of protein (30 μg) were then subjected to 10% SDS-PAGE, after which the separated proteins were transferred to onto polyvinylidene difluoride membranes (PVDF, Thermo Fisher Scientific). After first blocking the membranes in 5% nonfat dried milk in TBST for 1 h, they were incubated overnight at 4° C with the primary antibodies: anti-active caspase 9 (ab2324, 1:1000), anti-active caspase 3 (ab32042, 1:1000), anti-Bcl-2 (ab182858, 1:1000), anti-FOXO1 (ab179450, 1:1000), anti-p27 Kip1 (ab32034, 1:1000) and anti-GAPDH (ab9485, 1:1000). The membranes were then incubated for 1 h with HRP-labeled goat anti-rabbit secondary antibody (ab7097, 1:5000). Enhanced chemiluminescence (ECL) reagent (Thermo Fisher Scientific) was used to visualize the protein bands. Image J Software was used to quantify the intensity of the bands. All the antibodies were obtained from Abcam.

### Luciferase reporter assays

The FOXO1 3ʹ-UTR containing a putative miR-27a-3p binding site (FOXO1 WT 3ʹ-UTR) and a FOXO1 3ʹ-UTR with mutated binding site (FOXO1 MT 3ʹ-UTR) were synthetized by Genepharma (Shanghai, China) and cloned into pmirGLO vectors (Promega, Madison, WI, USA). The vectors (WT or MT) were then transfected into HT22 cells using Lipofectamine 2000 (Thermo Fisher Scientific), and the relative luciferase activity was detected using a dual luciferase reporter kit (Promega).

### *In vivo* experiment

Six-week-old Wistar rats were purchased from Vital River (Beijing, China) and housed within a dedicated specific pathogen-free facility. The rats were randomly divided into three groups: (1) sham-operated (Sham) (n=12); (2) I/R (n=12); and (3) I/R + miR-27a-3p agomir (n=12). To mimic CI/R *in vivo*, rats in groups (2) and (3) were anesthetized with 45 mg/kg sodium pentobarbital, and the middle cerebral artery (MCA) was occluded with a surgical filament. After 1 h of MCA occlusion, the filament was removed, and reperfusion proceeded. The rats in group (1) underwent the same procedure except for the filament insertion. Rats in groups (1) and (2) were injected with saline via intraventricular. Rats in group (3) were injected with miR-27a-3p agomir (RioboBio, Guangzhou, China) via intraventricular. At the end of the study, rats were sacrificed for the collection of brain tissues. Meanwhile, the apoptotic cells were detected by TUNEL staining as previously described [32].

At the end of the study, the rats were sacrificed, and the brain tissues were collected. Cerebral infarction area and cerebral water content were assessed. All *in vivo* experiments were performed in accordance with the U.S. National Institutes of Health Guide for the Care and Use of Laboratory Animals, following a protocol approved by the Ethics Committees of Zhejiang University School of Medicine.

### 2, 3, 5-Triphenyltetrazolium chloride (TTC) staining

In brief, the brains tissues from different groups were collected and frozen for 30 min at - 20° C. Then, the brain tissues were sliced into 2-mm-thick sections and incubated with 2% TTC solution (Sigma, St. Louis, MO, USA) at 37° C for 20 min, which was terminated by rinsing with PBS. Subsequently, the slice section was fixed with 4% paraformaldehyde for 2 h and photographed. The cerebral infarction area was expressed as a percentage of total infarct volume/total brain volume × 100%.

### Statistical analysis

All data are expressed as the mean ± standard error (S. D.). CCK-8 assays, immunofluorescence staining, western blotting, RT-qPCR, ELISAs, flow cytometry and ROS detection were performed in triplicate. In addition, all experiments were repeated three times. One-way analysis of variance (ANOVA) and post hoc Tukey’s tests were used for comparisons between at least three groups. Values of P<0.05 were considered statistically significant.

### Availability of data and materials

The datasets analyzed during the current study are available from the corresponding author on reasonable request.

### Ethics approval and consent to participate

All *in vivo* experiments were performed in accordance with National Institutes of Health guide for the care and use of laboratory animals, following a protocol approved by the Ethics Committees of Zhejiang University School of Medicine.
